# Remarks upon the Mortality of Exeter; Together with Suggestions Towards the Improvement of the Public Health. Being a Letter Addressed to Henery Hooper, Esq. the Right Worshipful the Mayor of Exeter

**Published:** 1845-10

**Authors:** 


ART. VI.—*Remarks upon the Mortality of Exeter; together with Suggestions towards the improvement of the Public Health. Being a Letter addressed to Henry Hooper, Esq. the right Worshipful the Mayor of Exeter.* By THOMAS SHAPTER, M.D. &c.—*London*, 1844.

THIS is one of the many useful publications to which the recent sanitary "agitation" has given birth. The author is already favorably known to the public by his work on the 'Climate of the South of Devon,' and the present essay cannot fail of adding to his reputation for science and humanity.

After presenting us with a short sketch of the mortality of Exeter, as compared with that of the country at large, of the surrounding rural districts, and of the large towns of England, he arrives at the conclusion, "that Exeter is not liable to a higher rate of mortality than is generally incident to favorably situated towns." It seems, indeed to occupy a sort of intermediate place between the two extremes of high and low mortality, but inclining toward the more favorable limit. Our author next glances at the views recently propounded by Mr. Neison, and presents us with tables of the ages of the living. He concluded this part of his essay by the following summary: 1. That Exeter, in common with other large cities, is liable to a greater amount of mortality than rural populations. 2. That the mortality of Exeter is very much in excess of that of the surrounding country. 3. That the average mortality of Exeter is less than that of the principal cities of England. 4. That there are but few cities of a similar amount of population, in which a lower rate of mortality prevails.

Dr. Shapter, however, is not satisfied with the comparatively favorable statement contained in the last proposition; but goes on to inquire—Has this city the amount of health and vitality which it ought to enjoy? This question receives the same answer which has been already given to the same question by all the large towns in England, nay, by every city in the world. Before entering into details with regard to the sanitary condition of Exeter, Dr. Shapter shows that its excess of mortality is not due to its situation:

"It must be recollected," he says, "that Devonshire, of which it is the chief city, is amongst the healthiest counties in the kingdom; that the district by which it is immediately surrounded, enjoys an average mortality less than that of any of the other districts of England, which the Registrar-general has selected for his Quarterly Reports; that the city is well placed as far as general position is concerned, situated on the ridges and crowning heights of elevated though sheltered ground; that it is built, for the most part, on a sandy soil, so that ventilation and drainage are not difficult; that the local provisions for drainage are extensive and well carried out; that the supply of water, whether natural or artificial, is ample; that a large and rapid river flows immediately beneath it; that the climate is mild and equable, neither over hot in summer, nor cold in winter; that the houses are not prominently ill constructed; that it contains within itself no destructive manufactures, nor deleterious employments; that it is, moreover, subject to no endemic disease, no ague, no particular fever; and that the medical relief for the poor is most ample. In fact, there is not, I believe, amongst the whole 115 districts of the Report, any one which presents so good an example of a town population uninfluenced by foreign causes, as does this city."

And yet Exeter can claim no exception from the common causes of disease and premature death which lurk within doors, in certain parts, at least, of all towns. In Exeter, as in London or Liverpool, we have examples of "families consisting of eight, ten, or more persons, having but the limited acommodation of a single room, ranging from ten to fourteen feet square, by nine feet high, in which they sleep by night, huddled together on beds and on the floor, and live by day, doing all the household work necessary for so large a number;"—of dame's schools held in rooms usually in crowded and bad situations, small, and inhabited by the mistress day and night, with the addition of a fire, winter and summer, for culinary purposes;—(in an atmosphere thus artificially heated, are congregated together for hours in the day, perhaps twenty children: the closeness and unpleasantness of these rooms is scarcely credible—they may really be termed modified "*black holes*;")—of pig-styes, and slaughter-houses, and crowded grave-yards;—of houses placed back to back, or with windows opening only on one side;—of inadequate provision of privies, of imperfect communication with the main sewers, and of other defects in the construction of their most important under-ground channels, &c. &c.

These defective sanitary arrangements are followed by their usual consequences, impaired health, and increased mortality. But we are informed that things are fast improving, that the sewerage is being attended to, the streets are being widened, and ample supply of pure water at a reasonable rate has been brought into the town.

Dr. Shapter concludes his essay by some simple recommendations which naturally spring out of the foregoing statements. He suggests "that as far as possible, blind alleys and small courts should be abolished; that slaughter-houses, and the keeping of animals should be prevented; that the streets should be opened, to admit the free passage of air; that the houses should be constructed with a view to perfect ventilation, adequately supplied with water and with sinks, &c., for necessary purposes; that the drains communicating with the sewers, as well as the sewers themselves, should be well constructed; that the embouchures of the latter should empty themselves below the level of the water, &c.; . . that holiday-ground should be provided for the poor and their children; that tepid and warm baths should be supplied at a moderate cost; and that for the richer citizens, detached houses should be built on healthy spots, at the distance of three or four miles from the city."

It was not to be expected that Dr. Shapter should discover any new remedy for the physical evils which he has pointed out. All these local reports must necessarily present much sameness. The story is getting an old and familiar one, but it must be told over and over again, till all men become familiar with it, and receive it as admitted truth. Though Dr. Shapter aims chiefly at the improvement of his own city, we have no doubt that his labours will be appreciated beyond its limits.

